# Cannabinoide reduzieren den Opioidverbrauch bei älteren Schmerzpatienten: Eine retrospektive Dreijahresauswertung einer ambulanten kassenärztlichen Praxis

**DOI:** 10.1007/s00482-022-00642-0

**Published:** 2022-04-06

**Authors:** K. Gastmeier, A. Gastmeier, F. Rottmann, T. Herdegen, R. Böhm

**Affiliations:** 1 Schmerzpraxis, Karl-Marx-Str. 42, 14482 Potsdam, Deutschland; 2 Facharztpraxis Bäkemühle, Zehlendorfer Damm 217, 14532 Kleinmachnow, Deutschland; 3 Institut für Experimentelle und Klinische Pharmakologie, Christian-Albrechts-Universität zu Kiel, Arnold-Heller-Straße 3, Haus U37, 24105 Kiel, Deutschland

**Keywords:** Endocannabinoidsystem, Cannabisextrakte, Geriatrie, Opioide, Schmerztherapie, Endocannabinoid system, Extracts, Geriatrics, Opioids, Pain therapy

## Abstract

**Hintergrund:**

Das Datenmaterial zur Verschreibung und therapeutischen Wirkung von medizinischen Cannabinoiden (CAM) im klinischen Alltag für ältere und geriatrische Patienten ist sehr beschränkt. Für diese Patienten rückt die Verordnung von CAM immer mehr in den therapeutischen Fokus.

**Ziel der Arbeit:**

Erfassung der Patientencharakteristika und Verordnung (Verordnungsdauer, Dosierung) von CAM (Dronabinol, Nabiximols, Cannabisextrakte) und komedizierten Opioiden einer schmerztherapeutischen Praxis.

**Methoden:**

Mit dem Stichtag 1. Juli 2020 wurde der Verbrauch von Opioiden (Morphinäquivalenz) und CAM-Tetrahydrocannabinol-Äquivalenz (THC-Äq.) für Männer bzw. Frauen und nach Alter analysiert.

**Ergebnisse:**

178 Schmerzpatienten wurden durchschnittlich (Median) 366 Tage (31 bis 2590 Tage) therapiert. Das Durchschnittsalter (Median) betrug 72 Jahre (26–96 Jahre); von den 115 Frauen (64,8 %) waren 34 jünger als 65 Jahre, 42 zwischen 65 und 80 Jahre und 40 über 80 Jahre alt; von den 63 Männern (35,2 %) waren 29 jünger als 65 Jahre, 24 zwischen 65 und 80 Jahre und 10 über 80 Jahre alt. Indikationen waren chronische Schmerzen und Einschränkungen der Lebensqualität. Von 1001 Verschreibungen waren 557 (55,6 %) Dronabinol als ölige Tropfen, 328 (32,7 %) Vollspektrumextrakte und 66 (6,6 %) Nabiximolsspray. 50 Rezepte (5 %) enthielten mehr als ein CAM simultan. Der Tagesverbrauch betrug im Median bei Dronabinolöl und Extrakten 9,6 mg THC, für Sprays 13,6 mg THC; er war bei Patienten > 64 Jahre konstant bzw. stieg bei jüngeren Patienten nichtsignifikant an. Frauen benötigten weniger THC als Männer (8,1 mg vs. 14,8 mg). 10 Patienten (5,6 %) brachen wegen fehlender Wirkung ab, 7 (3,9 %) wegen fehlender Kostenübernahme und nur 5 (2,8 %) wegen unerwünschter Arzneimittelwirkungen. 115 (65 %) Patienten erhielten gleichzeitig Opioide mit 65 Morphinäquivalenten/d im Median. Der Opioidverbrauch reduzierte sich signifikant um 24 Morphin-Äq./d (Median) bzw. 50 %, unabhängig von CAM-Dosis (< 7,5 oder > 7,5 mg THC-Äq./d), Geschlecht oder Alter.

**Diskussion:**

Schmerzpatienten profitieren von einer lang dauernden Therapie mit CAM, die sicher und signifikant auch in niedriger Dosis den Opioidverbrauch senken. Frauen benötigen evtl. weniger THC als Männer. Nebenwirkungen von THC limitieren nicht einen Therapieversuch mit CAM im höheren und hohen Alter.

## Abstract (en)

**Background:**

Relevant data for the prescription and therapeutic effects of medical cannabinoids (CAM) are still missing in everyday medicine especially for elderly and geriatric patients.

**Aim of the study:**

Documentation of prescription (duration, age) of CAM (dronabinol, nabiximols, cannabinoid extracts) and co-medicated opioids in a doctor’s office specializing in pain.

**Methods:**

Analysis of the consumption of opioids (morphine equivalent) and CAM (THC equivalent) for age and gender.

**Results:**

In all, 178 patients with chronic pain were treated for a period of 366 days (median; range 31–2590 days). Median age was 72 years (26–96 years); 115 were women (64.8%). Of these, 34 were younger than 65 years, 42 were 65–80 years and 40 were more than 80 years old. Of the 63 men, 29 were younger than 65 years, 24 were 65–80 years and 10 were older than 80 years. Indications for CAM were chronic pain and the limitations for opioids because of side effects and worsening of quality of life. To total of 1001 CAM were prescribed, 557 (55.6%) dronabinol as liquid, 328 (32.7%) as full spectrum extracts and 66 (6.6%) as oro-mucosal nabiximols spray. 50 prescriptions (5%) contained more than one CAM simultaneously. The daily consumption of dronabinol liquor and extracts were 9.6 mg/day (median), and of spray 13.6 mg. The dosage over time did not change in patients older than 64; in younger patients, there was a non-significant increasing trend. Women requested lower THC dosages compared to men (8.1 mg vs. 14.8 mg). Furthermore, 10 patients (5.6%) stopped CAM because of failing effectivity, 7 (3.9%) because of failing cost coverage and only 5 because of adverse side effects. 115 patients (65%) with CAM also received opioids a median 65 mg/day morphine equivalents. This opioid dosage was significantly reduced in course of time by 24 mg/day morphine equivalents or 50%. This reduction was independent on CAM dosage, age and gender.

**Discussion:**

Patients with chronic pain profit from long-term CAM which safely and significantly lower the consumption of comedicated opioids, even at low dosages (< 7.5 mg/day). For women, low-dose THC may be sufficient. Older patients benefit from CAM, and adverse effects do not limit the (chronic) use and prescription of CAM in the elderly.

Der Einsatz von CAM für die alltagsrelevanten Indikationen chronischer Schmerz und eingeschränkte Lebensqualität wird immer noch kritisch gesehen [7, 13, 28]. 2019 berichteten Wendelmuth et al. über einen substanziellen therapeutischen Nutzen von CAM für Ältere bei tolerablen Nebenwirkungen im ambulanten Praxisalltag. CAM bieten v. a. bei psychosomatischen Begleitreaktionen wie Schlaflosigkeit, Angst oder affektiver Verstimmung eine lohnende Therapieoption bei kalkulierbarem Nebenwirkungsrisiko [2, 11, 14, 15, 18, 31]. Bei der Nutzen-Risiko-Bewertung von CAM wird oft ignoriert, dass besonders Ältere bekanntermaßen unter den Nebenwirkungen von Analgetika und Neuropsychopharmaka leiden. Daher muss auch die Reduktion von Opioiden und Analgetika [22, 34] als ein wichtiger Therapieeffekt der CAM begriffen werden.

## Methoden

### Datenerhebung und Patienten

Diese Erhebung umfasst alle Patienten, die bis zum 30. Juni 2020 wegen eines Folgerezepts für ein CAM in die Sprechstunde kamen. Retrospektiv wurden bei 178 Patienten folgende Daten dokumentiert:

- Die Tage zwischen der Erstverordnung (durch die in der Praxis tätigen Autoren) und dem letzten Patientenkontakt im Beobachtungszeitraum,
- die Anzahl der bis dato ausgestellten BtM-Rezepte mit CAM und/oder Opioiden,
- die verordneten Mengen des jeweiligen CAM bzw. des jeweiligen Opioids,
- Therapieabbruch.

### Ethikvotum

Da die Patientendaten unmittelbar nach Extraktion anonymisiert wurden, war keine Beratung durch eine Ethikkommission erforderlich entsprechend § 15 Berufsordnung, wonach die Analyse anonymisierter Daten ohne vorherige Einwilligung möglich ist. Hier wurden die gleichen anonymisierten Daten analysiert, die für die gesetzlich vorgeschriebene Begleiterhebung erhoben werden mussten. In diese haben alle Patienten eingewilligt.

### Verordnung von CAM

Die Patienten wurden ausführlich über CAM aufgeklärt einschließlich über Wirkungen, die nach Inhalieren oder in hoher Dosis auftreten. Die Dosistitration begann mit der kleinstmöglichen Dosis, d. h. 1 Tropfen Dronabinol (entsprechend 0,8 mg THC), 0,1 ml Cannabisextrakt (entsprechend 1 oder 2,5 mg THC) oder einem Sprühstoß Nabiximols (entsprechend 2,7 mg THC).

Danach wurde langsam gesteigert bis zu einem ersten spürbaren Wirkungseffekt (Selbst- und/oder Fremdbeobachtung) oder belastenden Nebenwirkungen. Diese erstwirksame Dosis wurde nach Möglichkeit in der Folgezeit beibehalten, um mögliche Nebenwirkungen durch Dosissteigerung zu vermeiden. Die Verordnung erfolgte nach dem Therapieprinzip: „start low, go slow, stop hard and keep low“.

Therapieziel war nicht bzw. nicht nur eine prädefinierte Schmerzlinderung oder Appetitsteigerung, sondern auch die erste spürbare Verbesserung von psychoaffektiven Störungen wie Stress‑, Angst- und Erregungszuständen und/oder der Lebensqualität.

### Vergleichsgruppe

Als Vergleichsgruppe für die Veränderung der Opioiddosis unter CAM wurde aus dem gleichen Beobachtungszeitraum eine vergleichbare Kohorte unter den Praxispatienten herangezogen, die nur Opioide, aber keine CAM erhalten hatte. Diese Vergleichsgruppe umfasste 156 Patienten (im Median 71,3 Jahre, davon 96 Frauen), die sich weder in der Alter- und Geschlechtsverteilung noch in ihrer initialen Opioiddosis (50 vs. 65 Morphinäquivalente, *p* > 0,05; Abb. 5a) von der Beobachtungsgruppe unterschieden. Wie in der Beobachtungsgruppe auch war der überwiegende Teil der Diagnosen aus dem Bereich Krankheiten des Muskel-Skelett-Systems und des Bindegewebes und Krankheiten des Nervensystems.

### Statistik

Es handelt sich um eine rein deskriptive Studie ohne prädefinierte Ziele. Alle Ergebnisse sind explorativ und werden, wo eine Testung auf Unterschiede möglich war, mit unkorrigierten *p*-Werten angegeben.

Die Verteilung der Patienten nach Geschlecht, Alter und/oder Diagnose wurde mittels Chi-Quadrat-Tests geprüft.

Die täglichen CAM- oder Opioiddosen wurden mittels One-way-ANOVA zwischen den Gruppen verglichen. Gepaarte Vergleiche von Opioiddosierungen wie zwischen Beobachtungs- und Kontrollgruppe wurden mit dem zweiseitigen Student’s *t*-Test geprüft.

Intraindividuelle Veränderungen der Opioiddosis in einer Gruppe wurden mit einem einseitigen Student’s *t*-Test analysiert.

CAM-Dosierungen über den Therapieverlauf wurden mit dem Mann-Kendall-Test auf monotone Trends und mit dem Cox-Stuart-Test auf einen Drift untersucht.

## Ergebnisse

### Indikationen

Insgesamt wurden 178 Schmerzpatienten mit einem medianen Durchschnittsalter von 72 Jahren (Bereich 26 bis 96 Jahre) erfasst. Die durchschnittliche mediane Therapiedauer im Beobachtungszeitraum betrug 366 Tage (Bereich 31 bis 2590 Tage). Tab. 1 zeigt die Hauptindikation, die im Rahmen der Kostenübernahmebeantragung zu stellen war. Die meisten Patienten litten unter einer oder mehreren chronischen Erkrankungen, die Multimorbidität ist hier übersichtshalber nicht abgebildet.

**Table 1:** 

Gliederung	Titel	Anzahl	Prozent
G	Krankheiten des Nervensystems	79	44,5
M	Krankheiten des Muskel-Skelett-Systems und des Bindegewebes	73	41,0
C	Neubildungen	18	10,1
R	Symptome und abnorme klinische und Laborbefunde	4	2,2
B	Infektiöse und parasitäre Krankheiten	2	1,1
F	Psychische und Verhaltensstörungen	1	0,55
Q	Angeborene Fehlbildungen, Deformitäten und Chromosomenanomalien	1	0,55
Insgesamt	–	178	100,0

Im therapeutischen Fokus stand nicht eine Einzelindikation, sondern psychovegetative Störungen und Störungen der Lebensqualität, die auch als Störungen des Endocannabinoid(EC)-Systems gedeutet werden können [1, 6, 14, 15, 24]. Daher listet Tab. 1 nicht die den Antrag begründenden Diagnosen der Einzelindikation, sondern nur die Hauptgruppen der ICD-10 auf, denen die Einzelindikation zuzuordnen wäre. Weiterhin zielt diese Arbeit nicht auf eine Veränderung der Symptome ab, daher wird auf eine detaillierte Symptomdarstellung verzichtet. Die Verordnung von CAM basierte auf einem vermuteten dysfunktionalen EC-System, wie es bei konsumierenden Erkrankungen, affektiven und Angststörungen, Störungen der Stressachse oder Chronifizierungen beschrieben ist [1, 6, 24]. Verbindend für alle Patienten bzw. für den Einschluss in diese Erhebung waren die Schmerzen, wegen derer die Patienten unsere ambulante Schmerztherapiepraxis aufsuchten.

### Patientenpopulation

#### Patienten mit CAM.

**Figure 1:**
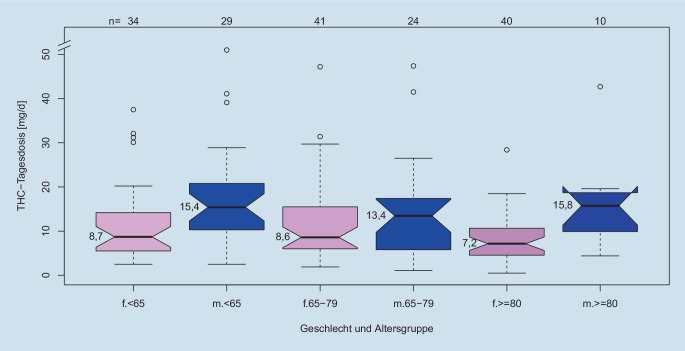

Von den 115 Frauen (64,8 %) waren 34 jünger als 65 Jahre, 42 zwischen 65 und 80 Jahre und 40 über 80 Jahre alt. Von den 63 Männern (35,2 %) waren 29 jünger als 65 Jahre, 24 zwischen 65 und 80 Jahre und 10 über 80 Jahre alt (Abb. 1).

Geschlecht und Alter waren ungleich verteilt, es gab signifikant mehr Frauen über 80 Jahre als erwartet (*p* = 0,02, Chi-Quadrat-Test; Tab. 2).

**Table 2:** 

		Weiblich	Männlich
		115	63
< 65 Jahren	63	34	29
65–79 Jahre	65	41	24
≥ 80 Jahre	50	40	10

#### Vergleichsgruppe ohne CAM.

Eine nach Altersgruppen und Geschlecht gleichartig verteilte Kontrollgruppe aus unserer Schmerzpraxis von 156 Schmerzpatienten mit Opioiden, aber ohne CAM wurde für die Vergleiche selektiert.

### Verbrauch von CAM nach Alter und Geschlecht

Die Mediane des THC-Tagesverbrauchs in den 3 männlichen bzw. 3 weiblichen Altersgruppen verteilten sich zwischen 5,6 mg und 11,1 mg pro Tag ohne signifikanten Unterschied. Männer benötigten deutlich mehr CAM als Frauen (14,8 mg vs. 8,1 mg), jedoch erlauben die unkorrigierten *p*-Werte keine Signifikanz. Entsprechend benötigten 44,7 % der Frauen (51 von 114), aber nur 25,4 % der Männer (16 von 63) 7,5 mg THC oder weniger.

Es gab einen nichtsignifikanten Trend für eine höhere CAM-Dosis in der Gruppe von jungen (< 65 Jahren) männlichen Patienten (*p* = 0,14, ANOVA) bzw. für eine niedrigere CAM-Dosis im Alter (Abb. 1).

### Verbrauch von CAM und Opioiden über die Zeit

**Figure 2:**
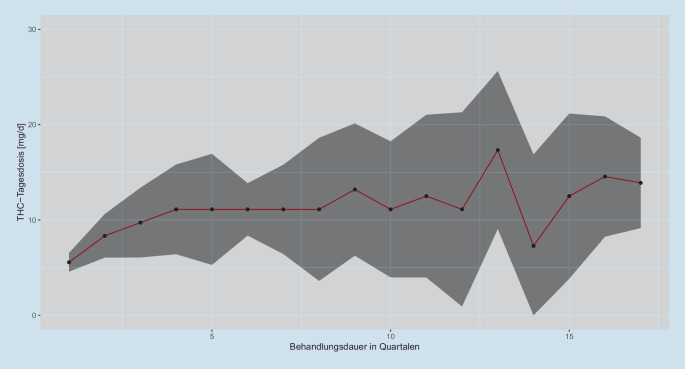

Der CAM-Verbrauch stieg signifikant über den Beobachtungszeitraum monoton an (*p* < 0,01, Mann-Kendall-Trendtest; Abb. 2), was vor allem durch die initiale Dosisfindung bedingt ist. Es gab aber keinen signifikanten Unterschied in der CAM-Dosis zu Beginn und Ende der Behandlung (*p* = 0,13, Cox-Stuart-Trendtest). Dies deutet auf eine stabile Dosierung über die Zeit nach der initialen Dosisfindung hin.

### Reduktion des Opioidverbrauchs in der Beobachtungsgruppe

**Figure 3:**
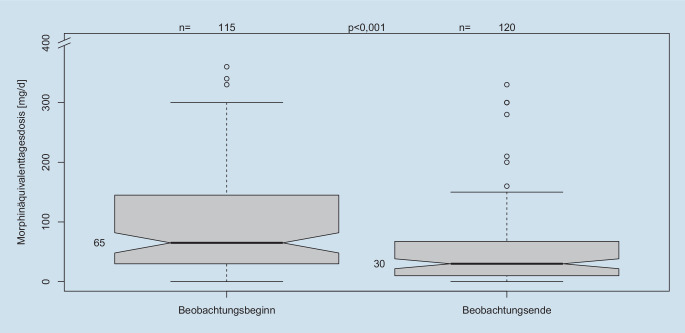

115 Patienten mit CAM aus der Beobachtungskohorte hatten zusätzlich ein Opioid in der Medikation. Zum Beobachtungsende hatten 120 Patienten ein Opioid (*p* = 0,74, Chi-Quadrat-Test mit Yates-Korrektur). Die Dosierungen zu Beginn und zum Ende waren mit 65 mg bzw. 30 mg Morphinäquivalenten im Median statistisch hochsignifikant unterschiedlich (*p* < 0,001, *t*-Test; Abb. 3).

**Figure 4:**
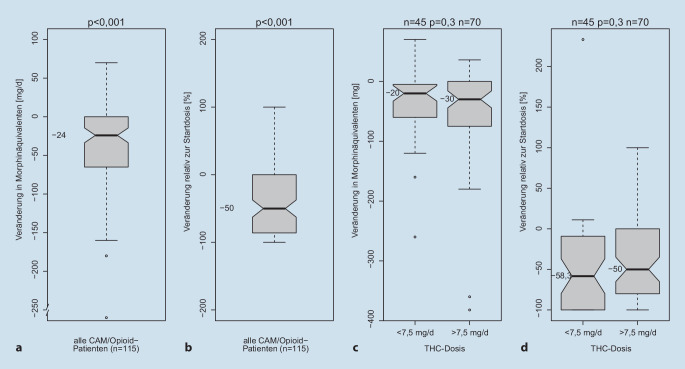

Während des Beobachtungszeitraums verminderte sich die individuelle Opioiddosis um 24 mg Morphinäquivalente, entsprechend 50 % der initialen Opioiddosis. Diese Reduktion war ebenfalls statistisch hochsignifikant (*p* < 0,001, einseitiger *t*-Test; Abb. 4a,b). Die Diskrepanz zwischen dem absoluten Wert der Morphinäquivalente und der intraindividuellen prozentualen Dosisreduktion ergibt sich aus der Verwendung von Medianen statt Mittelwerten, um Verzerrungen durch Ausreißer (z. B. sehr hohe Cannabinoiddosierungen) zu minimieren.

Die Reduktion der Opioiddosis war unabhängig von der CAM-Dosis. Es gab keinen Unterschied zwischen den niedrigen (< 7,5 mg/d THC) und höheren (> 7,5 mg/d THC) CAM-Dosierungen (*p* = 0,3, *t*-Test; Abb. 4c,d). Die Abnahme des Opioidverbrauchs korrelierte auch nicht mit anderen Parametern wie Alter, Geschlecht, Art des CAM oder der Indikation (Daten nicht gezeigt).

### Vergleich der Opioiddosierungsveränderung zwischen Beobachtungs- und Vergleichsgruppe

**Figure 5:**
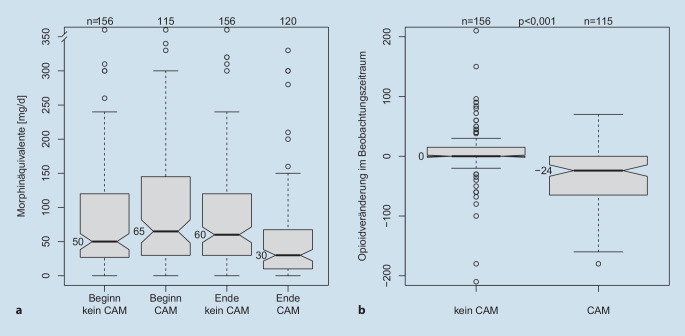

Die Opioidtagesdosen der bzgl. Alter, Geschlecht und Opioidverbrauch identischen Kontrollgruppe sowie der CAM-Beobachtungsgruppe zu Beginn und Ende der Beobachtung sind in Abb. 5a dargestellt. Der individuelle Opioidverbrauch änderte sich in der Kontrollgruppe nicht (Abb. 5b), aber nahm um −24 mg Morphinäquivalente in der CAM-Gruppe ab (Abb. 4a und 5b). Dieser Unterschied war zwischen den Gruppen statistisch hochsignifikant (*p* < 0,001, *t*-Test).

### Therapieabbrüche und unerwünschte Arzneimittelwirkungen

143 Patienten (80,3 %) führten die Therapie bis zum Ende des Beobachtungszeitraums fort. Abgebrochen wurde bei 10 (5,6 %) wegen fehlender Wirkung, bei 7 (3,9 %) wegen fehlender Kostenübernahme und nur bei 5 (2,8 %) wegen unerwünschter Arzneimittelwirkungen (Durchfall, kardiale Symptome, Kolik, Muskelkrämpfe; Tab. 3). Die Therapieabbrüche erfolgten im Median relativ spät nach 95 Tagen (31 bis 346 Tage).

**Table 3:** 

Behandlungsverlauf/Toleranz (*n*)			
143 (80,3 %)	Bleiben in Therapie (*n*)		
35 (19,7 %)	11 (6,2 %)	Abbruch: ineffektiv	
	10 (5,6 %)	Verstorben	
	7 (3,9 %)	Abbruch wegen fehlender Kostenübernahme	
	5 (2,8 %)	Abbruch wegen intolerabler UAW (nach im Median zwei Quartalen)	
		2 (1,1 %)	Diarrhö
		1 (0,6 %)	Kardiale UAW
		1 (0,6 %)	GIT-Spasmen
		1 (0,6 %)	Muskelkrämpfe
	2 (1,1 %)	Abbruch ohne weitere Angabe von Gründen	

## Diskussion

Diese Studie berichtet über den Langzeitverbrauch von CAM bei 178 Patienten (Durchschnittsalter im Median 72 Jahre) einer Schmerzpraxis, davon waren 50 Patienten (28,1 %) 80 Jahre und älter. Die durchschnittliche Therapiedauer betrug 366 Tage. Mehr als 80 % führten die CAM-Therapie bei konstanter CAM-Dosierung kontinuierlich fort. Zwei Drittel (66 %) der CAM-Patienten hatten eine Opioidkomedikation, deren Dosierung im Verlauf um ca. 50 % unabhängig von der komedizierten THC-Dosis reduziert werden konnte. Im Gegensatz dazu nahm die Opioiddosierung in der Kontrollgruppe (nur Opioide, keine CAM) nicht ab.

### Diagnostische Klassifikation der Patienten

Wir haben – ausgehend von der Hypothese einer Störung des ECS – unsere Patienten nicht nach Einzeldiagnosen mit konkreter ICD-10-Klassifikation, sondern nur nach ICD-Gliederungsgruppen zusammengefasst (Tab. 1). Angesichts der breiten THC-Wirkung (hohe kortikale und subkortikale Expressionsdichte des CB1-Rezeptors) erscheint die Einzelindikation von nachrangiger Bedeutung für eine CAM-Indikation. Wichtig ist den Autoren diejenige (niedrigste) Tagesdosis von CAM, mit der eine spürbare Linderung der chronischen oder konsumierenden Begleitsymptomatik bzw. eine spürbare Verbesserung der individuellen Lebensqualität erzielt werden kann.

### Verträglichkeit einer Langzeittherapie mit CAM

Nach einem initialen Anstieg blieb die Verordnungsdosis über die Zeit bei den über 65-Jährigen annährend konstant. Eine konstante Dosierung ohne die für Opioide typische Dosiserhöhung ist eine der Grundlagen der guten CAM-Verträglichkeit und bestätigt konstante THC-Wirkungen, die bei chronischen Schmerzpatienten in einem Beobachtungszeitraum von 48 Wochen beschrieben wurden [30].

Die Verträglichkeit war sehr gut, nur 5 Patienten beendeten die Einnahme von CAM wegen nichtschwerwiegender Nebenwirkungen, die aber mit einer deutlichen Latenz zur Einstellphase auftraten und in keinem zwingenden Kausalzusammenhang mit der CAM-Therapie standen.

Auch wenn diese Anwendungsbeobachtung aus dem Praxisalltag keine Placebokontrolle umfasst, ließ sich bei den Schmerzpatienten eine therapeutische Wirkung diagnostizieren, die eine Weiterverordnung sinnvoll erscheinen ließ. Ein Therapieabbruch von nur 5,6 % liegt weit unter den Abbruchraten der Langzeitverordnungen von Opioiden und COX-Hemmstoffen [10]. Die niedrigen Abbruchraten und die offensichtliche therapeutische Effektivität werden von der CAM-Begleiterhebung mit über 10.000 Datensätzen bestätigt [31].

Unsere Daten ermutigen zu einem Therapieversuch bei älteren bzw. sehr alten Patienten, auch wenn aus randomisierten, kontrollierten Studien bislang nur wenige Belege zur Wirksamkeit und Sicherheit von CAM bei dieser Personengruppe vorliegen [25, 32].

### Reduktion der Opioiddosis

Eine synergistische Wirkung von Cannabinoiden und Opioiden sowie der opioidsparende Effekt der CAM werden seit Langem diskutiert und beschrieben [11, 18, 26]. Wendelmuth et al. [34] haben eindrucksvolle Opioideinsparungen unter CAM beschrieben und substanzielle Symptomverbesserungen, die entweder auf die CAM und/oder auf die Reduktion der Opioide zurückgeführt werden können. Die aktuelle Anwendungsbeobachtung, die auch 35 Patienten über 80 Jahre der Studie von Wendelmuth et al. [34]. umfasst, erweitert diese Ergebnisse bezüglich des Beobachtungszeitraums von bis zu 3 Jahren. Die Opioiddosis konnte signifikant um 50 % zur Ausgangsdosis bzw. 24 Morphinäquivalente reduziert werden. Da die unerwünschten Opioidwirkungen oft dosisabhängig sind, ermöglicht eine solche Reduktion eine bessere Verträglichkeit der chronischen Schmerztherapie. Im Gegensatz dazu nahm die Opioiddosis in der praxiseignen Kontrollgruppe (keine CAM) nicht ab.

Zahlreiche präklinische Befunde berichten über eine Dosisminderung von Opioiden durch CAM. Im Tierversuch reduzierte THC dramatisch die Menge an Opioiden im Vergleich zu einer Analgesie unter Opioiden allein [22]. Als ein möglicher Mechanismus gilt die Heterodimerisierung von MOR und CB1-Rezeptoren und eine Verstärkung der Signaltransduktion [17, 36]. Schließlich beeinflusst das Endocannabinoidsystem das Endorphinsystem und die Responsivität auf Opioide [3].

Ein wesentlicher Befund dieser Anwendungsbeobachtung ist die Reduktion des Opioidverbrauchs bereits bei CAM-Niedrigdosierungen, die ebenso effektiv war wie die höheren Dosierungen.

### Wirksamkeit niedriger CAM-Dosierungen

Der mediane THC-Tagesbedarf von 9,75 mg („range“ 0,5 bis 175,7 mg/d) ist dem niedrigen Dosisbereich zuzuordnen bei einem Patientengut, das repräsentativ für unsere Praxis ist. Niedrige THC-Dosierungen wirken subjektiv stressreduzierend, während höhere THC-Dosen über 12,5 mg die negative Stimmung erhöhen können [8, 20]. Bei spastischen Schmerzen wurde mit 1 mg/d Nabilon (entspricht ca. 7,5 bis 8 mg/d THC) eine signifikante Schmerzreduktion erreicht [35], bei neuropathischen Schmerzen und Fibromyalgie verbesserten 7,5 mg/d (5–12,5 mg) THC als Add-on-Medikation signifikant die Lebensqualität und reduzierten sowohl Schmerz wie die primäre Medikation [33]. Schließlich war bei opioidrefraktären Tumorschmerzen niedrig dosiertes Nabiximolsspray (1 bis 4 Sprühstöße pro Tag) einer täglichen Hochdosistherapie mit 11 bis 16 Sprühstößen sowohl bei der Schmerzreduktion als auch bei Schlafstörungen und Lebensqualität signifikant überlegen.

Die Bedeutung einer Niedrigdosistherapie bei Älteren wird unterstützt von Befunden, dass niedrig dosiertes THC in alten Mäusen neuroprotektiv und kognitionsverbessernd wirkt [5] sowie zirkadiane und Lebensrhythmen günstig beeinflusst [16]. Für die Einordnung niedrig dosierter CAM in die „evidence-based medicine“ gilt die Aussage von Lötsch et al. [21]: „While controlled studies showed a lack of robust analgesic effects, cannabis was nearly always associated with analgesia in open-label or retrospective reports, possibly indicating an effect on well-being or mood rather than on sensory pain“ (deutsche Übersetzung: „Während kontrollierte Studien einen Mangel an deutlichen analgetischen Wirkungen zeigen, war Cannabis in offenen oder retrospektiven Studien fast immer mit einer Analgesie assoziiert; das zeigt möglicherweise eher eine Wirkung auf Wohlbefinden oder Stimmung als einen Effekt auf sensorischen Schmerz“). Und besonders das zählt im schmerztherapeutischen und palliativmedizinischen Alltag.

### Frauen und Niedrigdosis-THC

Besondere Aufmerksamkeit gilt dem Befund, dass 44,7 % der Frauen, aber nur 25,4 % der Männer eine Niedrigdosis < 7,5 mg benötigen, über alle Altersgruppen hinweg nahmen Frauen deutlich weniger THC als Männer (8,1 mg vs. 14,8 mg). Diese eindeutigen Daten unserer Beobachtung bestätigen Studien, dass Frauen für die gleiche Wirkung signifikant weniger THC benötigen [23] bzw. im Niedrigdosisbereich von 5 mg signifikant stärker auf THC respondieren als Männer [9]. Es stellt sich die klinisch relevante Frage, inwieweit bei Frauen die erhöhte Sensitivität für THC mit der erhöhten Vulnerabilität einer gestörten Angstverarbeitung einhergeht.

### Wirtschaftliche Bedeutung der Niedrigdosistherapie von CAM

Die Empfehlungen zur wirtschaftlichen Verordnungsweise von CAM der Kassenärztlichen Bundesvereinigung von 3/2019 arbeiten die Problematik einer CAM-Therapie an Einzeldiagnosen ab ohne pathophysiologischen Bezug zum dysfunktionalen Endocannabinoidsystem (ECS). Auch Literaturrecherchen fokussieren auf Einzelindikation [12], ihre Ergebnisse zeigen oft geringe Evidenz bzw. viele Nebenwirkungen bei hohen Dosen. So berichtet eine Metaanalyse zur indikationsbezogenen Schmerztherapie sehr hohe Tagesdosen von 32 bis 130 mg THC/d [27] oder 200 mg/d THC zur Vorbeugung von Migräne [29]. Im Gegensatz dazu berichten die Schmerztherapeuten übereinstimmend über Effekte zwischen 7,5 und 15 mg THC im Steady-State bei nichtgeriatrischen Patienten (Praxisleitlinien von Gottschling et al. [11] und Horlemann et al. [18]), diese Dosierungen entsprechen auch der Begleiterhebung [31].

Calabrese [4] stellte fest: „Viele Forscher konzentrieren sich nicht auf die in ihren Tabellen und Abbildungen angegebenen Reaktionen bei niedrigen Dosen, sondern entscheiden sich dafür, nur die Wirkungen unter hohen Dosierungen zu betrachten.“ Dies wird sehr oft den Erfordernissen bzw. „medical needs“ im praktischen schmerztherapeutischen Alltag nicht gerecht. Dazu passt die zusammenfassende Feststellung von Karst [19]: „Cannabinoide lindern in einer ausgeprägten individuellen Unterschiedenheit Schmerzen; … Begleitsymptome von Schmerzen wie Stress und Schlafstörungen werden positiv beeinflusst; … es gibt einen Opioideinspareffekt; … für klinisch bedeutsame Effekte reichen oft geringe Dosierungen.“

## Fazit für die Praxis

- Unsere Studie zeigt einen positiven Effekt einer THC-Niedrigdosis bei chronisch und/oder krebskranken, älteren wie sehr alten Patienten über lange Zeiträume. Zur guten Verträglichkeit bei diesem durch Nebenwirkungen häufig belasteten Patientengut kann die signifikante substanzielle Opioidreduktion beitragen, auch im THC-Niedrigdosis-Bereich. Unsere Therapiestrategie „start low, go slow, stop hard“ bei den Zeichen der ersten Symptombesserung hat sich im Praxisalltag bewährt. Um dem Prinzip des „non nocere“ gerecht zu werden, muss die Forderung nach Ausschöpfung oft riskanter Therapiealternativen zugunsten eines früheren Einsatzes von (niedrigen) CAM geändert werden.

## References

[1] Bennett MR, Arnold J, Hatton SN, Lagopoulos J (2017). Regulation of fear extinction by long-term depression: the roles of endocannabinoids and brain derived neurotrophic factor. Behav Brain Res.

[2] Bialas P, Drescher B, Gottschling (2019). Cannabispräparate bei chronischen Schmerzen: Indikationen, Präparateauswahl, Wirksamkeit und Sicherheit : Erfahrungen der saarländischen Schmerztherapeuten. Schmerz.

[3] Bruehl S, Burns JW, Morgan A (2019). The association between endogenous opioid function and morphine responsiveness: a moderating role for endocannabinoids. Pain.

[4] Calabrese EJ (2008). Hormesis and medicine. Br J Clin Pharmacol.

[5] Calabrese EJ, Rubio-Casillas A (2018). Biphasic effects of THC in memory and cognition. Eur J Clin Invest.

[6] Chanda D, Neumann D, Glatz JFC (2019). The endocannabinoid system: overview of an emerging multi-faceted therapeutic target. Prostaglandins Leukot Essent Fatty Acids.

[7] Chang-Douglass S, Mulvihill C, Pilling S, Guideline Committee (2020). Cannabis-based medicinal products: summary of NICE guidance. BMJ.

[8] Childs E, Lutz JA, de Wit H (2017). Dose-related effects of delta-9-THC on emotional responses to acute psychosocial stress. Drug Alcohol Depend.

[9] Fogel JS, Kelly TH, Westgate PM, Lile JA (2017). Sex differences in the subjective effects of oral ∆^9^-THC in cannabis users. Pharmacol Biochem Behav.

[10] Gehling M, Hermann B, Tryba M (2011). Meta-analysis of dropout rates in randomized controlled clinical trials: opioid analgesia for osteoarthritis pain. Schmerz.

[11] Gottschling S et al (2018) Expertenkonsens – Medizinischer Einsatz von Cannabinoiden. Lehre & Praxis, Heft 9, ISSN 2199-3564

[12] Häuser W, Finn DP, Kalso E (2018). European Pain Federation (EFIC) position paper on appropriate use of cannabis-based medicines and medical cannabis for chronic pain management. Eur J Pain.

[13] Häuser W, Petzke F, Fitzcharles MA (2018). Efficacy, tolerability and safety of cannabis-based medicines for chronic pain management—An overview of systematic reviews. Eur J Pain.

[14] Herdegen T (2020). Pharmako-legendär: Cannabis – zwischen Dichtung und Wahrheit. Dtsch. Apoth. Z..

[15] Herdegen T (2020). Pharmako-legendär: Cannabis – Pharmakologie. Dtsch. Apoth. Z..

[16] Hodges EL, Ashpole NM (2019). Aging circadian rhythms and cannabinoids. Neurobiol Aging.

[17] Hojo M, Sudo Y, Ando Y (2008). mu-Opioid receptor forms a functional heterodimer with cannabinoid CB1 receptor: electrophysiological and FRET assay analysis. J Pharmacol Sci.

[18] Horlemann J et al DGS-Praxisleitlinie. Cannabis in der Schmerztherapie. 2018. Version: 1.0 für Fachkreise. www.dgs-praxisleitlinien.de/index.php/leitlinien/cannabis

[19] Karst M Chronische Schmerzen – Wie und wann wirkt Cannabis? • allgemeinarzt-online. http://www.allgemeinarzt-online.de. Zugegriffen: 4. Okt. 2020

[20] Lafenêtre P, Chaouloff F, Marsicano G (2007). The endocannabinoid system in the processing of anxiety and fear and how CB1 receptors may modulate fear extinction. Pharmacol Res.

[21] Lötsch J, Weyer-Menkhoff I, Tegeder I (2018). Current evidence of cannabinoid-based analgesia obtained in preclinical and human experimental settings. Eur J Pain.

[22] Maguire DR, France CP (2014). Impact of efficacy at the μ-opioid receptor on antinociceptive effects of combinations of μ-opioid receptor agonists and cannabinoid receptor agonists. J Pharmacol Exp Ther.

[23] Matheson J, Sproule B, Di Ciano P, Fares A, Le Foll B, Mann RE, Brands B (2020). Sex differences in the acute effects of smoked cannabis: evidence from a human laboratory study of young adults. Psychopharmacology.

[24] Mendiguren A, Aostri E, Pineda J (2018). Regulation of noradrenergic and serotonergic systems by cannabinoids: relevance to cannabinoid-induced effects. Life Sci.

[25] Minerbi A, Häuser W, Fitzcharles MA (2019). Medical cannabis for older patients. Drugs Aging.

[26] Nielsen S, Sabioni P, Trigo JM (2017). Opioid-sparing effect of Cannabinoids: a systematic review and meta-analysis. Neuropsychopharmacology.

[27] Petzke F, Enax-Krumova EK, Häuser W (2016). Wirksamkeit, Verträglichkeit und Sicherheit von Cannabinoiden bei neuropathischen Schmerzsyndromen: Eine systematische Übersichtsarbeit von rando-misierten, kontrollierten Studien. Schmerz.

[28] Petzke F, Karst M, Gastmeier K, Radbruch L, Steffen E, Häuser W (2019). Ad-hoc-Kommission der Deutschen Schmerzgesellschaft „Cannabis in der Medizin“. Ein Positionspapier zu medizinischem Cannabis und cannabisbasierten Medikamenten in der Schmerzmedizin [Position paper on medical cannabis and cannabis-based medicines in pain medicine. Schmerz.

[29] Pressetext: Cannabinoide zur Vorbeugung von Migräne geeignet. https://www.pressetext.com/news/20170624001. Zugegriffen: 13. Dez. 2020

[30] Schimrigk S, Marziniak M, Neubauer C (2017). Dronabinol is a safe long-term treatment option for neuropathic pain patients. Eur Neurol.

[31] Schmidt-Wolf G, Cremer-Schaeffer P (2021). 3 Jahre Cannabis als Medizin – Zwischenergebnis der Cannabisbegleiterhebung. Bundesgesundheitsbl.

[32] Volicer L, Stelly M, Morris J (1997). Effects of dronabinol on anorexia and disturbed behavior in patients with Alzheimer’s disease. Int J Geriatr Psychiatry.

[33] Weber J, Schley M, Casutt M (2009). Tetrahydrocannabinol (delta 9‑THC) treatment in chronic central neuropathic pain and fibromyalgia patients: results of a multicenter survey. Anesthesiol Res Pract.

[34] Wendelmuth C, Wirz S, Torontali M (2019). Dronabinol bei geriatrischen Schmerz- und Palliativpatienten: Eine retrospektive Auswertung der ambulanten kassenärztlichen Therapie. Schmerz.

[35] Wissel J, Haydn T, Müller J (2006). Low dose treatment with the synthetic cannabinoid Nabilone significantly reduces spasticity-related pain: a double-blind placebo-controlled cross-over trial. J Neurol.

[36] Zador F, Receptome WM (2015). Interactions between three pain-related receptors or the „Triumvirate“ of cannabinoid, opioid and TRPV1 receptors. Pharmacol Res.

